# Low-melting point agarose as embedding medium for MALDI mass spectrometry imaging and laser-capture microdissection-based proteomics

**DOI:** 10.1038/s41598-023-45799-5

**Published:** 2023-10-31

**Authors:** Francesco Greco, Luca Fidia Pardini, Asia Botto, Liam Andrew McDonnell

**Affiliations:** 1 Institute of Life Sciences, Sant’Anna School of Advanced Studies, Pisa, Italy; 2 Fondazione Toscana Gabriele Monasterio, Pisa, Italy; 3 Fondazione Pisana per la Scienza ONLUS, San Giuliano Terme (PI), Italy; 4 Department of Chemistry and Industrial Chemistry, University of Pisa, Pisa, Italy

**Keywords:** Bioanalytical chemistry, Imaging, Mass spectrometry, Proteomic analysis

## Abstract

The combination of MALDI mass spectrometry imaging, laser-capture microdissection, and quantitative proteomics allows the identification and characterization of molecularly distinct tissue compartments. Such workflows are typically performed using consecutive tissue sections, and so reliable sectioning and mounting of high-quality tissue sections is a prerequisite of such investigations. Embedding media facilitate the sectioning process but can introduce contaminants which may adversely affect either the mass spectrometry imaging or proteomics analyses. Seven low-temperature embedding media were tested in terms of embedding temperature and cutting performance. The two media that provided the best results (5% gelatin and 2% low-melting point agarose) were compared with non-embedded tissue by both MALDI mass spectrometry imaging of lipids and laser-capture microdissection followed by bottom-up proteomics. Two out of the seven tested media (5% gelatin and 2% low-melting point agarose) provided the best performances on terms of mechanical properties. These media allowed for low-temperature embedding and for the collection of high-quality consecutive sections. Comparisons with non-embedded tissues revealed that both embedding media had no discernable effect on proteomics analysis; 5% gelatin showed a light ion suppression effect in the MALDI mass spectrometry imaging experiments, 2% agarose performed similarly to the non-embedded tissue. 2% low-melting point agarose is proposed for tissue embedding in experiments involving MALDI mass spectrometry imaging of lipids and laser-capture microdissection, proteomics of consecutive tissue sections.

## Introduction

MALDI mass spectrometry imaging (MSI) is a technique that allows for spatial mapping of various analytes including lipids^1^, peptides^2^, metabolites^3^ and glycans^4^. The technique is often applied to biological tissues to investigate the molecular composition of heterogeneous specimens^5^ or to perform a segmentation of the tissue to highlight characteristics that are not detectable by histology alone^6^. In order to increase proteome coverage tissue regions can be excised and isolated by laser-capture microdissection (LCM) and then analyzed with proteomics workflows^7^.

In the context of the MALDI MSI-guided microdissection, highly reproducible sectioning across consecutive sections is essential to ensure that regions identified by MALDI MSI can be reliably isolated from subsequent tissue sections (i.e. the sectioning and mounting preserves the longitudinal make-up of the sample). Formalin-fixed paraffin-embedded (FFPE) tissue samples are the preferred format for histopathological analysis^8^ because of the high quality sections that can be routinely obtained (essential for many established diagnostic pathways), as well as their compatibility with immunohistochemical molecular assays and the fact that the tissues may be stored at room temperature.

FFPE tissue blocks are routinely used for MALDI–MSI of proteolytic peptides and enzyme-released N-linked glycans^9,10^, but are less suited to the analysis of lipids and metabolites because the tissue processing leads to the loss of some lipids/metabolites, and the long processing times lead to post-mortem changes in the molecular composition of the tissue metabolome. Note that some lipids and metabolites have been detected from FFPE tissue sections, and which may also have diagnostic potential^11,12^, but the loss of metabolites and post-mortem changes make it less straightforward to link the metabolic signatures to the tissue’s underlying biology. For these reasons fresh-frozen tissue samples are recommended for MALDI-MSI of metabolites and lipids, but the fragility of fresh-frozen tissue sections makes the sectioning more challenging, especially for high fat content tissues.

The embedding of fresh frozen tissue within an inert matrix provides structural support and thereby aids sectioning. The most common embedding medium is optimal cutting temperature (OCT) compound, a polymer that has excellent thermal properties and produces a dense embedding that allows for easy sectioning over a wide range of temperatures. OCT is widely used in immunohistochemistry, histology, and immunofluorescence. However, the high ionization efficiency of its polymeric components cause heavy ion suppression in MALDI MSI^13,14^. Protocols have been reported for the removal of OCT prior to protein^15^ and lipid^16^ MALDI-MSI analysis, and alternative embedding media have been developed.

The most desirable characteristic of an embedding medium is that it should provide structural stability to the sample without interfering with the analysis, i.e. providing the same result as non-embedded samples. Nelson et al*.*^17^ conducted an extensive investigation of the compatibility of many embedding media with MALDI MSI, to embed fragile zebrafish samples, selecting the one that provided the best cutting performance and gave no interference in MSI analysis. However, the comparison was limited to only MALDI MSI. Dannhorn et al*.*^18^ investigated the properties of several embedding media and their compatibility with MSI techniques (MALDI-MSI, desorption-electrospray ionization (DESI), secondary-ion mass spectrometry (SIMS), and imaging mass cytometry (IMC)), as well as with immunohistochemistry, RNAseq, and genomics analysis, providing an excellent starting point for multimodal studies. Nonetheless, the evaluation of the media compatibility with proteomics analysis was not performed. Quanico et al.^19^ focused on the integrated MALDI MSI, lipidomics and proteomics analysis from the same tissue section. They were able to perform both MALDI MSI and proteomics from 2% gelatin and 2% carboxymethyl cellulose (CMC) embedded samples, but they did not focus on the comparison of the two media nor performed a comparison with non-embedded samples.

The ideal embedding medium is a material that does not affect the analysis, providing similar results to that obtained from non-embedded samples. All the previous examples, despite providing extensive characterization of embedding media, did not benchmark the results to non-embedded tissues. Such benchmarking is essential in order to perform a cost-vs-benefit assessment, and thereby select the experimental procedure best suited to the goals of the experiment.

Here we report an investigation of low-temperature embedding media in terms of their mechanical properties and embedding temperature, with the goal of determining a reliable embedding medium for the combination of MALDI MSI of lipids, laser-capture microdissection of tissue regions, and quantitative proteomics. We compared embedded and non-embedded tissues to ensure that the embedding media did not interfere with the analysis. 2% low-melting point agarose and 5% gelatin-embedded were selected as optimal embedding media, and enabled the collection of high-quality consecutive sections. While 5% gelatin gave a light ion-suppression effect in MALDI-MSI experiment, 2% low-melting point agarose did not interfere with neither MALDI MSI nor the quantitative proteomics experiments, producing results similar to the non-embedded samples. 2% low-melting point agarose is thus proposed as embedding medium for MALDI MSI of lipids and proteomics of microdissected tissue samples from consecutive tissue sections.

## Results and discussion

An optimal embedding medium should enable the production of high-quality consecutive sections, allowing for the artifact-free spatially resolved analysis of fragile samples and the isolation of these regions from consecutive sections. Previously reported embedding media for MALDI MSI include carboxymethyl cellulose (CMC)^19,26^, gelatin^17,19^, agarose^17^, HPMC-PVP (hydroxypropyl methylcellulose and polyvinylpyrrolidone mixture)^18^, and poly[N-(2-hydroxypropyl)methacrylamide] (pHPMA)^27^.

Here we focused on CMC, gelatin and low- melting point agarose since these media are relatively inexpensive and have previously been reported to provide excellent characteristics (structural support and low chemical interference) for MALDI MSI investigations. For instance, a combination of 2% CMC and 10% gelatin was selected by Nelson et al*.* as the optimal embedding medium for the sectioning of fragile zebrafish embryo samples^17^. Low melting point agarose is a type of agarose used for nucleic acid electrophoresis, with a gel temperature a few degrees above room temperature^28,29^. It was included in the study as a protein-free embedding medium. Here we evaluated seven combinations of the embedding media in terms of embedding temperature, cutting performance, compatibility with MALDI MSI of lipids, LCM, and quantitative proteomics.

### Embedding temperature and cutting performance evaluation

It is known that freeze–thaw cycles can adversely affect MALDI-MSI and proteomics analysis of tissue samples^30^. To minimize the number of freeze–thaw cycles experienced by the tissue samples it would be better to perform the sample embedding-and-freezing directly after tissue collection, however such a process would extend the post-mortem time at room temperature and is not always logistically practical or possible. In these cases, an embedding medium with low embedding temperature is recommended to minimize sample thawing, especially for small samples. The embedding temperature was evaluated for the embedding media listed below, together with their cutting performance:

- 2% CMC
- 2% CMC + 5% gelatin
- 2% CMC + 10% gelatin
- 5% CMC + 1% low melting point agarose
- 5% gelatin
- 1% low melting point agarose
- 2% low melting point agarose

These tests were performed by embedding chicken breast tissue in the selected media. The embedding temperature was measured with a digital thermometer and defined as the temperature of medium gelatinization. Embedded samples were then sectioned to test their cutting properties. The embedded blocks were equilibrated at – 20 °C in the cryostat and 5, 8 and 12 μm thick sections were collected. All embedding media tested allowed the collection of consecutive tissue sections, with little difference between the conditions. Denser media (e.g. 2% CMC + 5% gelatin and 2% CMC + 10% gelatin) performed slightly better during cutting, producing more solid sections that were easier to manipulate and to mount on the glass microscope slides. A summary of these results is provided in Table 1.

**Table 1:** Comparison of embedding temperature and cutting performance of the embedding media.

Embedding medium	Embedding temperature (°C)	Cutting performance	Overall performance
2% CMC	< 3	Good	Medium
2% CMC + 5% gelatin	24	Very good	Poor
2% CMC + 10% gelatin	20	Very good	Medium
5% CMC + 1% low melting point agarose	28	Very good	Poor
5% gelatin	17	Good	Optimal
1% low melting point agarose	16	Satisfactory	Good
2% low melting point agarose	20	Good	Optimal

Overall performance is based on embedding temperature i.e. the temperature of medium gelatinization, cutting performance (capability of producing high-quality consecutive sections) and ease of medium preparation, including solubilization time.

2% CMC showed good cutting performance, allowing the production of consecutive tissue sections. When the embedding temperature was tested, the medium appeared to freeze instead of gelatinizing at temperatures lower than 3 °C, remaining liquid at 4 °C. A similar behavior was previously reported by Quanico et al., who noted that 2% CMC liquefies after thaw mounting sections of CMC-embedded tissues on parafilm-coated glass slides^19^. This property is not ideal for the preparation of precast gels^31^, or for the fabrication of two-pieces gel molds for the embedding of small samples^32^. Despite good cutting performance, the embedding temperature of the other CMC-containing media was in general higher than the other tested media. The higher gel temperature increased the embedding time and increased the risk of tissue degradation. Moreover, CMC was more difficult to solubilize and significantly increased the preparation time. In contrast 5% gelatin and 2% low melting point agarose were easy to solubilize and had low gelatinization temperatures, ensuring rapid sample freezing and minimal tissue degradation. The low-melting/freezing temperature of agarose meant that it remained liquid at room temperature, therefore allowing lower embedding temperatures. The cutting performance of 5% gelatin and agarose-based media were good and allowed for the collection of consecutive tissue sections. The 5% gelatin and 2% low-melting point agarose were selected for further investigation, with regards to their compatibility with mass spectrometry analysis.

### MALDI MSI of lipids of embedded tissues

An optimal embedding medium for MALDI MSI should not interfere with the molecular signals obtained from the tissue, both in terms of distribution and detection sensitivity. To test media compatibility with MALDI MSI of lipids, mouse liver samples were embedded with 5% gelatin and 2% low-melting point agarose, analyzed, and compared with non-embedded mouse liver tissue. Fractional mass plots were obtained for the three conditions to highlight the presence of background signals arising from the embedded tissues.

**Figure 1:**
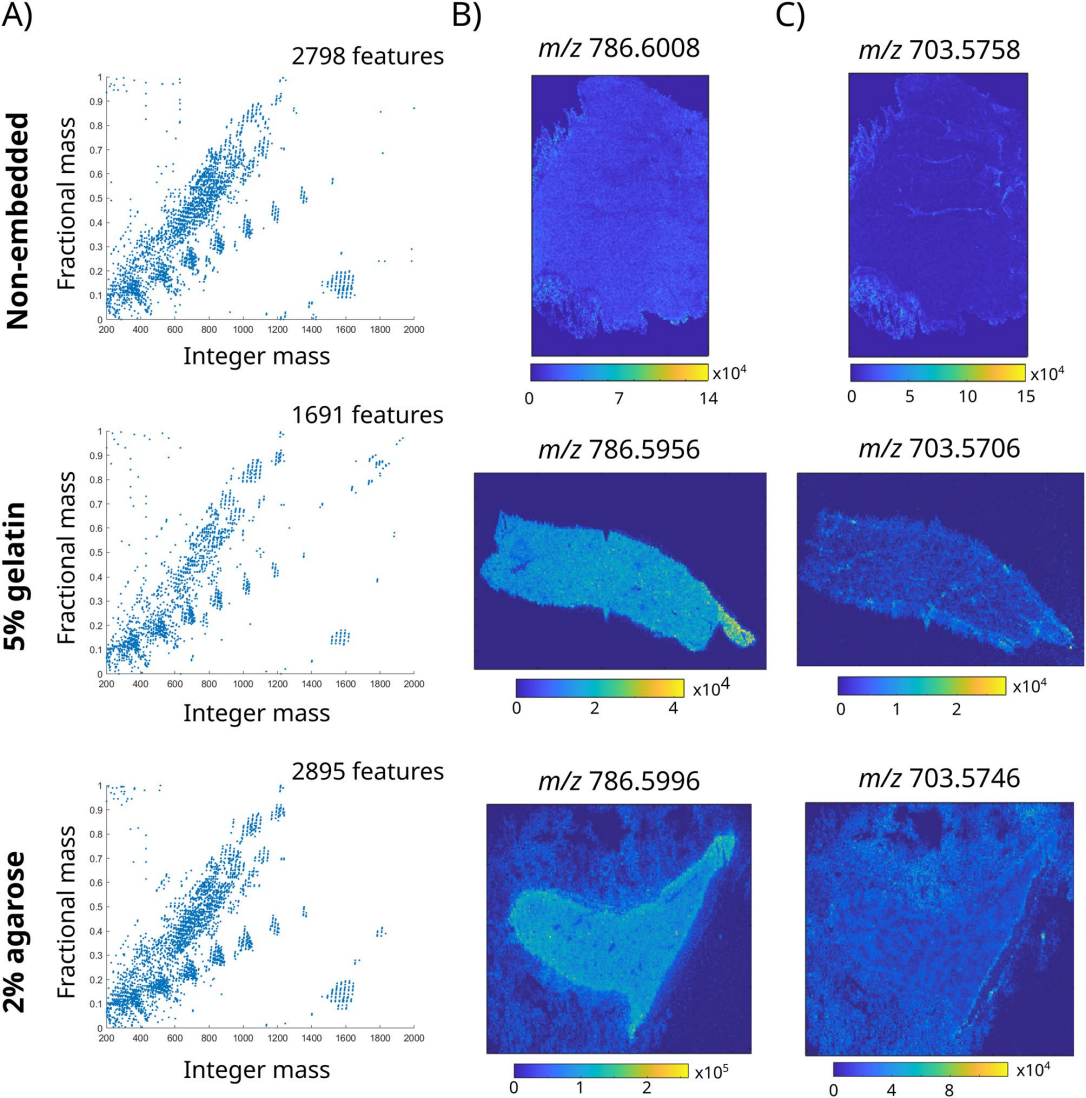
Comparison of MALDI MSI data acquired from liver samples embedded in 5% gelatin, 2% agarose, and non-embedded samples. (**A**) Fractional mass plots of the mean mass spectra acquired from the 5% gelatin, 2% agarose embedded samples, and from the non-embedded samples. (**B**,**C**) show the distributions of two lipid ions, assigned to proton adducts of PC(36:2)/PE(39:2) and SM(34:1)/PE-Cer(37:1).

The comparison between MALDI MSI data of non-embedded samples, 5% gelatin, and 2% low-melting point agarose embedding media is summarized in Fig. 1. Both media provided good quality tissue blocks that allowed the collection of consecutive sections and proved to be MALDI-MSI compatible, with no traces of contamination in the MALDI mass spectra. The fractional mass plot of the average mass spectrum for both embedding media (Fig. 1A) showed a large number of molecular ions with masses between 700 and 800 *m/z* on the integer axis and 0.3–0.7 *m/z* on the fractional mass axis, corresponding to lipid signals^33^. The comparison of fractional mass plots showed that samples embedded with 5% gelatin produced fewer lipid peaks compared to both agarose and the non-embedded samples.

Molecular ion images obtained from the agarose-embedded tissue samples exhibited higher background signals outside the tissue section compared to the gelatin-embedded and non-embedded tissue (Fig. 1B,C) but the fine lobular structure of the liver observable in the non-embedded tissue was also still recognizable for both agarose-based embedding media (Fig. 1C). Signal arising from the background is likely to come from the embedding, which was not removed prior to MSI analysis. The retention of the fine structure in the MSI images indicates that the background signal is not due to delocalization of the lipid signals into the embedding media. Both 5% gelatin and 2% agarose embedding media allowed high quality sections to be obtained, from which good quality MALDI MSI datasets could be recorded. Despite the higher background outside the tissue obtained with the 2% agarose embedding media, the MALDI-MSI data better matched that obtained from the non-embedded tissue in terms of the appearance of the fractional mass plot, and the lobular structure of the liver in the MALDI-MSI images.

### Evaluation of LCM-bottom-up microproteomics performance of embedded tissues

The compatibility of the gelatin and agarose embedding media with proteomics of microdissected tissue samples was tested by processing small 2 mm^2^ microdissected tissue samples with the SP3^21^ proteomics protocol.

**Figure 2:**
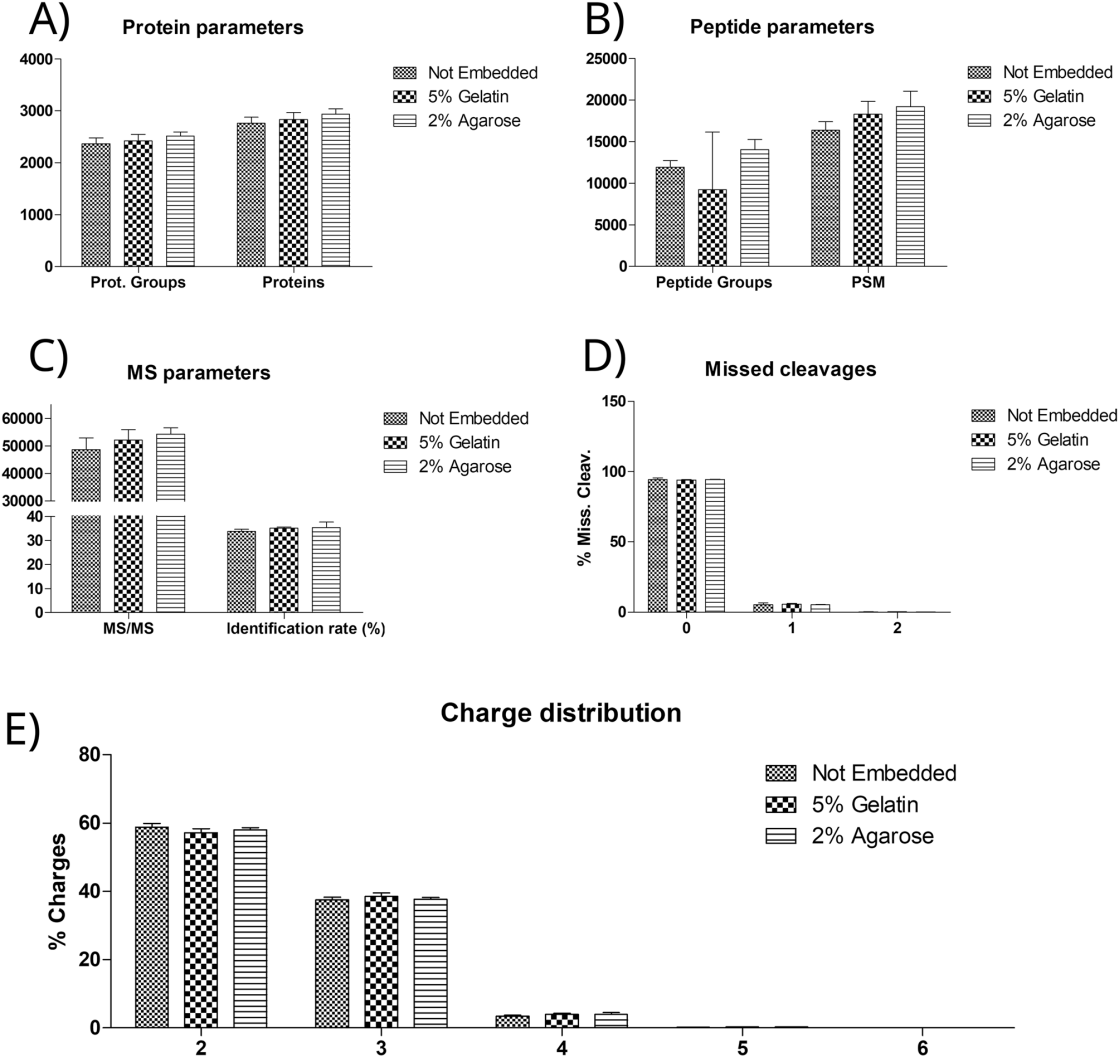
Comparison of the number of identified proteins and protein groups (**A**), peptide groups and PSMs (**B**), MS/MS spectra and identification rate (**C**), missed cleavages (**D**), and the charge state distribution (**E**).

Figure 2 shows a comparison of the number of identified protein groups, identified proteins, identified peptide groups, peptide-spectrum matches (PSMs), and acquired mass spectra acquired, as well as the identification rate for the three embedding conditions. PSMs are the MS^2^ spectra that were assigned to a peptide, including redundancies. Identification rate was calculated as the ratio between PSMs and total number of MS^2^ spectra. The percentage of PSMs with zero, one and two missed cleavages sites and the charge distribution of PSMs was also compared to evaluate the digestion efficiency (Supplementary Information S1).

A one-way ANOVA analysis of the proteomics digestion quality parameters showed no significant difference between the three embedding conditions. The number of proteins groups, proteins, peptides and PSMs identified from 5% gelatin and 2% agarose embedded tissue did not significantly differ from non-embedded tissue. The number of acquired MS/MS spectra and the identification rate was also not significantly different, demonstrating that the embedding did not introduce any significant difference in the number of detected peptides, even for the 5% gelatin embedding medium. Type I and type II porcine collagen, which are expected to be major components of porcine skin gelatin, were not identified in any of the samples, confirming the absence of gelatin contamination from the embedding medium. The percentage of PSMs with zero, one and two missed cleavage sites and the charge state distribution were identical for the three different conditions, suggesting that the embedding had no effect on digestion efficiency.

**Figure 3:**
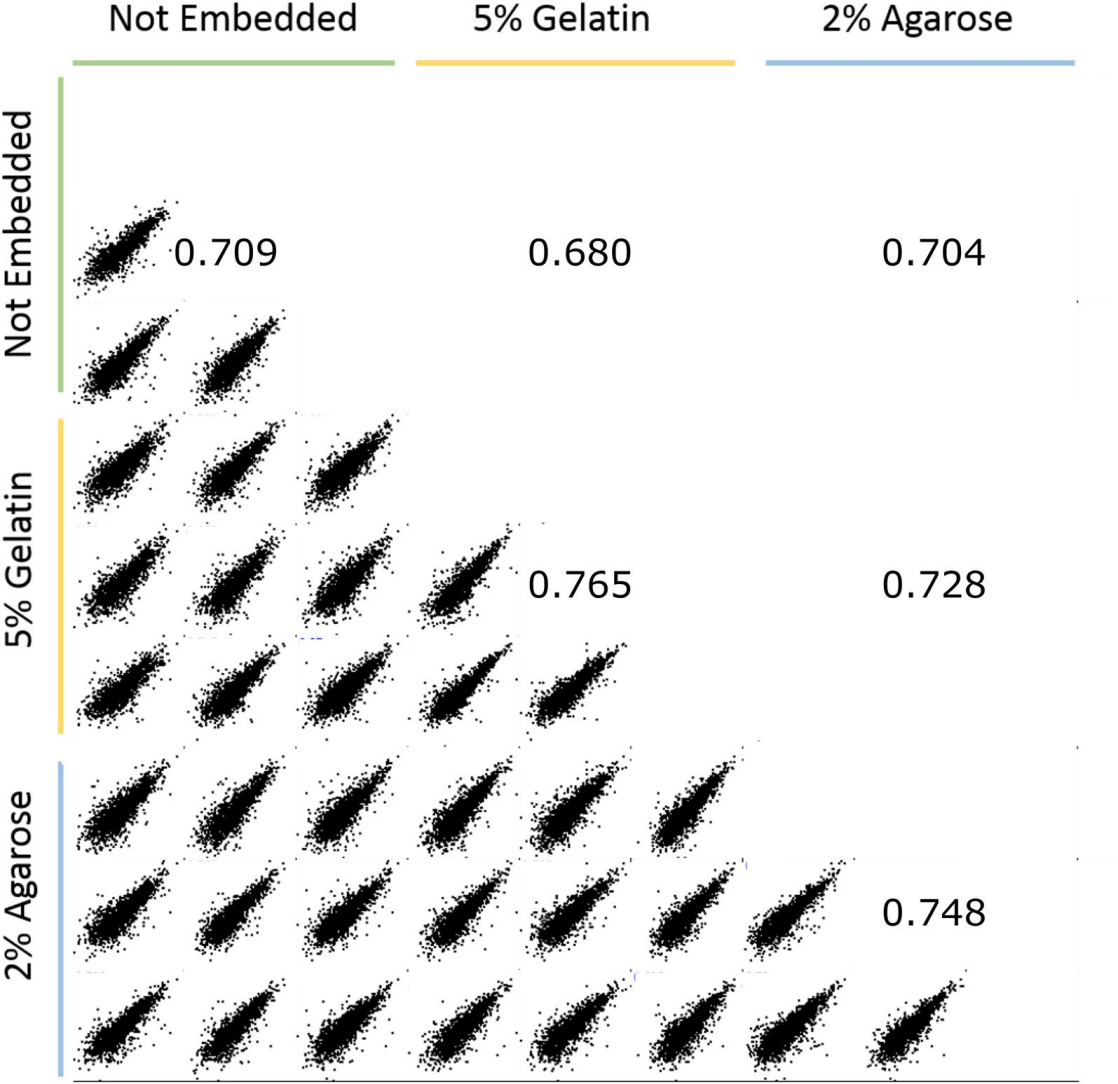
Multiscatter plot of protein intensities. Upper right part of the matrix reports the average Pearson correlation coefficient.

The effect of the embedding medium on protein quantification was also investigated. A table containing the quantification of all proteins in the samples is provided in Supplementary Information S2. Figure 3 shows multiscatter plots depicting the pairwise comparison of the three embedding conditions. The upper right half of the correlation matrix shows the average Pearson correlation coefficient.

The average Pearson correlation coefficient of the protein intensities revealed that the protein intensities obtained from the 2% agarose embedded tissue were a closer match to the non-embedded tissue than the gelatin embedded tissue (0.704 vs 0.680 respectively).

## Discussion

The central tenet behind multimodal imaging *using consecutive tissue sections* is the conservation of the biologically relevant spatio-molecular information between consecutive tissue sections. Nonetheless, the sectioning of fragile and heterogeneous tissues can complicate such multimodal approaches, since the collection of superimposable sections can be challenging. Embedding media help to overcome this disadvantage by facilitating the collection of reproducible consecutive sections.

OCT is the most widely used embedding medium for fresh-frozen samples. However, when samples are to be analysed by mass spectrometry, OCT is not recommended, since it is acts as a contaminant causing ion suppression^13,14^. This is particularly apt for the SP3 methodology, which is based on the adsorption of proteins and peptides onto bead surfaces (OCT is based on polydiethylene glycol, a surfactant, which can interfere with this adsorption). Tissue washes have been developed to reduce this contamination^15,16^, and alternative embedding media have been developed to enable MALDI-MSI based multimodal approaches. Nonetheless, a complete characterization of embedded media which included a comparison with non-embedded tissue was missing. Here we focused on the use of combinations of CMC, gelatin and low-melting point agarose as mass spectrometry-compatible media. The embedding temperature, the cutting performance, and the compatibility with MALDI MSI of lipids and LCM-proteomics were evaluated.

Among all the embedding media tested, 5% gelatin and 2% low melting point agarose allowed for low-temperature embedding, optimal cutting performances and mass spectrometry compatibility compared to non-embedded sample. In fact, no contamination was observed in the MALDI MSI of lipids experiment. The comparison of the fractional mass plots of MSI data of embedded samples with non-embedded tissue suggested a potential ion-suppression effect of gelatin embedding. Instead, the number of lipids identified in the 2% agarose-embedded sample was comparable with the non-embedded tissue. Another potential advantage of the agarose-based embedding medium over the gelatin-based medium is the possibility of performing on-tissue tryptic digestion for MALDI MSI of peptides. The 2% agarose medium produced a higher background outside the tissue but this phenomenon had no effect on the ion distribution recorded inside the tissue. In fact, the absence of ion delocalization is confirmed by the retention of the spatial features of the sample (in our case, the fine lobular structure of the liver). The signal surrounding the tissue is likely to be produced by the ionization of the agarose embedding, which was not removed before MSI data acquisition. Hematoxylin and eosin H&E staining of consecutive tissue sections showed that the agarose-based embedding remained attached to the slide during staining. Nevertheless, the background of the 2% agarose embedding in the H&E staining was negligible (Supplemental Fig. S1). Both gelatin and agarose were found to be compatible with laser-capture microdissection and proteomics and the embedding medium did not influence the number of identified proteins nor the quality of protein digestion. The protein intensities obtained from tissues embedded in 2% low melting point agarose had a slightly higher correlation coefficient with those obtained from not embedded tissue, compared to 5% gelatin.

Based on these results, both 5% gelatin and 2% low-melting point agarose were suitable embedding media for LCM-proteomics experiments. Nonetheless, low-melting point agarose outperformed gelatin in MALDI MSI analysis of lipids. 2% low-melting point agarose showed good performances for the embedding of frozen biological samples for multimodal experiment involving MALDI MSI of lipids and proteomics of excised regions from consecutive tissue sections.

## Conclusions

Mass spectrometry-based multimodal spatial experiments are based on the assumption of the conservation of the spatial details between consecutive sections. This assumption is no longer valid if the reproducibility across the section collection is not maintained, due to the introduction of distortion during tissue sectioning and mounting. Embedding media are crucial to ensure proper section collection and to collect reproducible sections.

An ideal embedding medium should provide structural stability to the tissue and should not interfere with the molecular analysis i.e. producing the same results as the non-embedded tissue. Thus benchmarking with non-embedded tissue is crucial for the evaluation of embedding media. While several MALDI MSI-compatible embedding media have been proposed^17,18^, only 2% CMC and 2% gelatin were also tested for proteomics analysis^19^, but which were not benchmarked against non-embedded tissue. Here we reported the use of 5% gelatin and 2% low-melting point agarose for the embedding of fresh-frozen samples, MALDI MSI of lipids, microdissection, and quantitative proteomics. The comparison of the results obtained from the embedded tissues with the non-embedded samples demonstrated little adverse effects of the embedding process, limited to just light ion-suppression effect of gelatin in MALDI-MSI experiments. 2% low-melting point agarose is thus proposed as embedding medium for the acquisition of MALDI MSI of lipids and proteomics of microdissected tissue samples from consecutive tissue sections.

## Materials and Methods

### Materials

CMC (C9481-500G, carboxymethylcellulose sodium, medium viscosity) and gelatin (48723-500G-F) were purchased from Sigma-Aldrich (St. Gallen, Switzerland); QA-Agarose low melting point was obtained from MP Biomedical, Santa Ana, CA, USA. Disposable PVC plastic embedding moulds (15 × 15 × 5 mm) were obtained from Polysciences (Warrington, PA, USA). HPLC grade water and DMSO (dimethyl sulfoxide) were purchased from Thermo Fisher Scientific (Dreieich, Hessen, Germany). Histology grade ethanol was purchased from DiaPath S.p.A. (Martinengo, Italy). Harris hematoxylin was purchased from VWR International LLC (Milan, Italy), cOmplete Protease Inhibitor Cocktail was purchased from La Roche (Basel, Switzerland), and Trypsin/Lys-C mix Mass Spec grade was obtained from Promega (Milan, Italy).

ITO (indium tin oxide) coated glass slides for MALDI were purchased from Bruker Daltonics (Billerica, MA, USA), and the polyethylene naphthalate (PEN) slides and Adhesive Cap 200 were purchased from Zeiss (Oberkochen, Germany). Speedbeads A (GE65152105050250), Speedbeads B (GE65152105050250), 1M HEPES (4-(2-hydroxyethyl)-1-piperazineethanesulfonic acid) solution, EDTA (ethylenediaminetetraacetic acid), EGTA (ethylene glycol-bis(β-aminoethyl ether)-*N,N,N′,N′-*tetraacetic acid), sodium dodecyl sulphate (SDS), trifluoroethanol (TFE), dithiothreitol (DTT), iodoacetamide (IAA), formic acid, acetonitrile (ACN) and HPLC grade ethanol were purchased from Sigma-Aldrich (Milan, Italy).

### Methods

#### Sample embedding

Fresh frozen mouse liver (male C57BL/6, adult) was kindly provided by M. Caleo at the Institute of Neuroscience of the CNR in Pisa and stored at – 80 °C until use. Chicken breast was purchased from a local supermarket, cut into 1 cm × 1 cm × 0.5 cm pieces and stored at – 80 °C until use.

Embedding solutions were prepared by dissolving the powdered compound in HPLC grade water and heating the resulting solution to 95 °C. A dry-ice ethanol bath was prepared, and the lower part of the embedding mold was submerged into the bath. Embedding media were cooled down to a temperature just above their hardening temperature. A small piece of frozen tissue was then placed in the mold and the embedding medium carefully added with a plastic pipette, avoiding the formation of bubbles. After the medium had solidified the mold was transferred on dry ice, wrapped in aluminum foil and stored at -80 °C. Tissue blocks were sectioned using a Leica CM1950 cryostat (Leica, Wetzlar, Germany). Tissue sections of 12 μm thickness were mounted onto indium-tin-oxide (ITO) conductive glass slides for MALDI MSI experiments. 12 μm thick sections were also collected on polyethylene naphthalate (PEN) membrane glass slides for microdissection. Non embedded mouse liver tissue was sectioned alongside the embedded samples, to benchmark the results obtained from the embedded samples. The cryostat blade was cleaned with ethanol after the collection of each tissue section to avoid cross contamination.

#### MALDI MSI

Tissue sections mounted on ITO glass slides were thawed under vacuum for 15 min prior to matrix application. One replicate was processed for each condition. The MALDI matrix norharmane (7 mg/mL in CHCl_3_:MeOH 70:30) was sprayed onto the tissue sections using a SunCollect automated spraying system (SunChrom, Friedrichsdorf, Germany). X and Y deposition speed were set to Low 3 and Medium 1 respectively while the height of the nozzle was set to 50 mm. The matrix solution was applied in 8 layers (2 layers at 5 µL/min followed by 6 layers at 10 µL/min). The matrix coated tissue section was then dried under vacuum for 15 min prior to data acquisition.

MALDI MSI was performed using an elevated pressure (EP) MALDI source^20^ (Spectroglyph, LLC., Kennewick, WA, USA) equipped with a 349 nm laser (Spectra-Physics, Santa Clara, CA, USA), coupled to an Orbitrap Q-Exactive Plus mass spectrometer (Thermo Scientific). The laser was operated at 1.65 A and 500 Hz, the source pressure was 7.2 Torr and the pixel size was 30 × 30 µm. Mass spectra were acquired in the range 150–2000 *m/z* at 70 000 resolving power at *m/z* 200. The position file was aligned to the raw MS files using ImageInsight (v. 0.1.0.11550, Spectroglyph, LLC).

#### Laser-capture microdissection and quantitative proteomics

Tissue sections were stained with hematoxylin at 4°C and all solutions used for staining contained a tablet of protease inhibitor. The LCM system used was an Apotome2 Axio Observer Z 1 microscope equipped with a PALM MicroBeam (Zeiss, Oberkochen, Germany). PEN slides were mounted on the LCM system and the laser used to isolate around 2 mm^2^ of homogeneous liver tissue. The dissected tissue pieces were collected using RoboLPC (Robo laser pulse catapulting) mode, which works by cutting the selected region border and catapulting the dissected tissue piece into the cap of an Adhesive Cap 200 tube with a defocused laser pulse.

Each tissue sample was processed in triplicate using an SP3 proteomics workflow^21,22^. In brief, rectangular tissue regions were transferred from the adhesive cap to 0.2 mL tubes containing 10 μL of SP3 lysis buffer and 10 μL TFE. Samples were lysed by sonication and quantified using a modified BCA assay suited for small sample amounts^23^. Aliquots of 1.95 μg of proteins were prepared and the sample volume was equalized to 40 μL with 1:1 SP3 lysis buffer:TFE. 2 μL of carboxylate coated paramagnetic beads (100 mg/mL suspension of 50% Speedbeads A and 50% Speedbeads B) were added to each sample. Proteins were denatured at 95 °C for 5 min, then reduced with the addition of 2 μL of 200 mM DTT and incubated at 45°C for 30 min. Proteins were alkylated with the addition of 2 μL of 400 mM IAA followed by incubation at room temperature for 30 min after which the reaction was quenched by the addition of 2 μL of 200 mM DTT. The solution was then acidified by the addition of 2 μL of formic acid, and ACN added to a final concentration of 50% to promote protein adsorption to the magnetic beads. The tubes were then placed on a magnet to immobilize the magnetic beads and the bead-bound proteins were washed with EtOH 70% and ACN 100%. The proteins were eluted from the beads using 10 μL 50mM HEPES pH 8, and with sonication (30 s ON, 30 s OFF, 5 min, 18 °C) to help protein detachment. The proteins were then digested overnight (18 h, trypsin/Lys-C mix Mass Spec grade, 1:25 enzyme/protein). The resulting proteolytic peptides were then purified: the ACN concentration was raised to 95% to promote peptide adsorption to the beads, which were then washed with 100% ACN and finally eluted using 20 μL of 2% DMSO aqueous solution.

Peptide samples were diluted 1:1 with 10% formic acid and injected into the EASY-nLC 1000 coupled to an Orbitrap Fusion mass spectrometer (Thermo Scientific, Bremen, Germany). Peptide ions were analyzed using the Top Speed data-dependent method, with a 3 s cycle; MS1 scans were performed in the Orbitrap (m/z 375 to 1500 at 120 K resolution with an AGC Target 5 × 10^5^ and 100 ms maximum injection time) and MS2 scans were acquired in the ion trap using a 1.6 *m/z* isolation window, 30% HCD Collision Energy, and an AGC target of 5 × 10^3^.

#### Data analysis

##### MALDI MSI

Thermo RAW file were converted into mzXML format using RawConverter^24^ and the tool ORBIIMAGEmzXML2Tricks (v.0.10, G. Eijkel) was used to convert the mzXML spectra file and the EP-MALDI XML position file into a .mat data structure. The MALDI MSI dataset was TIC normalized.

To evaluate the number and nature of detected peaks, the fractional mass plot of the *m/z* list of each MALDI MSI dataset was plotted. The fractional mass plot is a visualization method based on the decomposition of the ion *m/z* values into their integer and decimal components. The fractional mass plot was obtained by plotting the integer part as the *x* coordinate and the decimal part as the *y* coordinate.

##### LC–MS/MS

LC–MS/MS data were analyzed using the Proteome Discoverer (v.2.1, Thermo Fisher Scientific, Rockford, IL, USA) and searched against the SwissProt Mus Musculus database (Uniprot, 11 June 2019, 17,021 entries). An in-house contaminant database was added to the search (253 entries), which included type I (alpha-1 and alpha-2 chains) and type III (alpha-1) porcine collagen. Searches were performed with a precursor mass tolerance of 10 ppm using a strict FDR of 0.01. A maximum of two missed cleavages were allowed. Methionine oxidation (+ 15.995 Da) were set as a dynamic modification and carbamidomethylation of cysteine (+ 57.021 Da) was set as a static modification. Quantification was performed only on proteins identified by at least one unique peptide; proteins identified from the contaminant database were discarded. Protein intensities were used as a proxy of protein abundance. The proteomics data were analyzed using Perseus 1.5 software^25^ and GraphPad Prism v5 for Windows (GraphPad Software, La Jolla, CA, USA, www.graphpad.com). The number of identified protein groups, proteins, peptide groups, peptide-spectrum-matches (PSM), and mass spectra were retrieved from Proteome Discoverer software. Identification rate was defined as the percentage ratio of PSM to mass spectra. The distribution of peptide charge and the percentage of missed cleavages were retrieved. Identification parameters (number of identified protein groups, proteins, peptide groups, peptide-spectrum-matches (PSM), mass spectra, identification rate, percentages of missed cleavages and charge distribution) were compared using a one-way ANOVA performed with using (α = 0.05). Protein intensities were log2 transformed and the median of protein intensity of each sample was subtracted from each protein intensity to correct for small differences in the number of peptides loaded on the LC–MS/MS instrument. Perseus software was used to produce a multiscatter plot (based on Pearson’s correlation coefficient) to compare the protein intensities obtained from the technical replicates and from the different embedding media/control.

### Supplementary Information

Supplementary Information 1.

Supplementary Information 2.

Supplementary Information 3.

## Data Availability

The MALDI MSI and the proteomics data have been deposited to the ProteomeXchange Consortium via the PRIDE^34^ partner repository with the dataset identifier PXD045349. (https://proteomecentral.proteomexchange.org/cgi/GetDataset?ID=PXD045349).
