# Supplementary material for: Low-melting point agarose as embedding medium for MALDI mass spectrometry imaging and laser-capture microdissection-based proteomics
Source: Sci Rep. 2023 Oct 31;13:18678. doi: 10.1038/s41598-023-45799-5 (PMC10618491; doi:10.1038/s41598-023-45799-5)
Supplement: Supplementary file 3 — Supplementary Information 3. [file 41598_2023_45799_MOESM3_ESM.docx]

Low-melting point agarose as embedding medium for MALDI mass spectrometry imaging and laser-capture microdissection-based proteomics

Francesco Greco^1,2,3^, Luca Fidia Pardini^4,3^, Asia Botto^4,3^, Liam A. McDonnell^3^

1. Institute of Life Sciences, Sant’Anna School of Advanced Studies, Pisa, Italy;

2. Fondazione Toscana Gabriele Monasterio, Pisa, Italy

3. Fondazione Pisana per la Scienza ONLUS, San Giuliano Terme (PI), Italy;

4. Department of Chemistry and Industrial Chemistry, University of Pisa, Pisa, Italy

Corresponding author:

Liam Andrew McDonnell

[liam@fpscience.it](mailto:liam@fpscience.it)

**Supplemental Information**

**
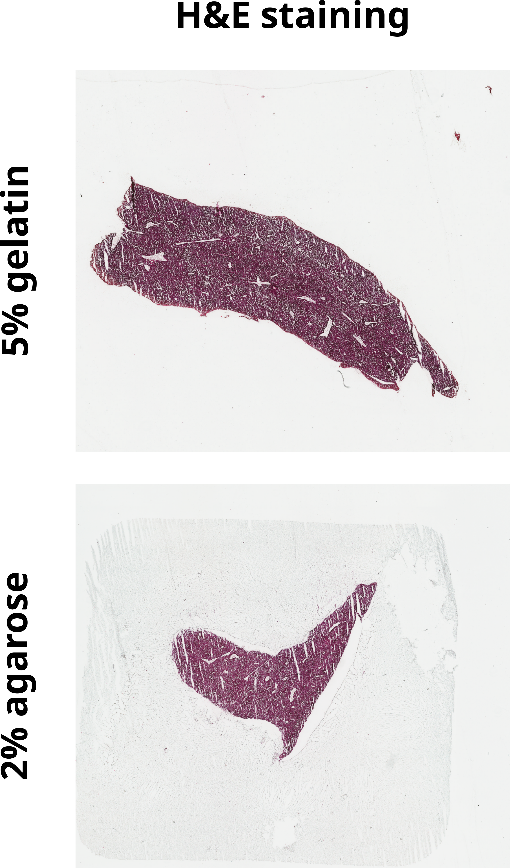
**

**Supplemental Figure 1**. Optical images of 5% gelatin and 2% agarose- embedded sections after hematoxylin and eosin staining. 5% gelatin embedding detached from the slide during the staining; the 2% agarose medium remained attached to the slide but produced only a negligible background.

**Supplemental Information 1.** Summary of the protein and peptide identification parameters of the proteomics comparison of non-embedded, 5% gelatin and 2% low-melting point agarose embedded tissue.

**Supplemental Information 2**. Quantification table of the results of the proteomics comparison of non-embedded, 5% gelatin and 2% low-melting point agarose embedded tissue.
